# Motiv8: a study protocol for a cluster-randomised feasibility trial of a weight management intervention for adults with severe mental illness in secure forensic services

**DOI:** 10.1186/s40814-024-01458-8

**Published:** 2024-03-02

**Authors:** Rebekah Carney, Hany El-Metaal, Heather Law, Siobhan Savage, Ingrid Small, Mark Hann, Gemma Shields, David Shiers, Parise Carmichael-Murphy, Richard Jones, Elizabeth Kimber, Andrew McDonald, Sophie Parker

**Affiliations:** 1 Youth Mental Health Research Unit, Greater Manchester Mental Health NHS Foundation Trust, Manchester, UK; 2 Division of Psychology and Mental Health, University of Manchester, Manchester, UK; 3 Greater Manchester Mental Health NHS Foundation Trust, Manchester, UK; 4 Division of Population Health, Health Services Research, and Primary Care, School of Health Sciences, University of Manchester, Manchester, UK; 5 Psychosis Research Unit, Greater Manchester Mental Health NHS Trust, Manchester, UK; 6 School of Medicine, University of Keele, Staffordshire, UK; 7 Lancashire and South Cumbria, NHS Foundation Trust, Preston, UK; 8 School of Nursing and Midwifery, University of Bolton, Bolton, UK

**Keywords:** Forensic, Randomised controlled trial, Lifestyle, Physical health intervention, Mental health

## Abstract

**Introduction:**

People with severe mental illness have physical comorbidities which result in significant reductions in quality of life and premature mortality. Effective interventions are required that are suitable for people in secure forensic mental health services. We conducted pilot work of a multidisciplinary weight management intervention (Motiv8) which showed improvements in physical and mental health and high levels of satisfaction. We aim to test the feasibility of Motiv8 under cluster randomised conditions, with an aim to investigate the acceptability, feasibility and potential effectiveness of this intervention to supplement standard secure care.

**Methods and analysis:**

A randomised waitlist-controlled feasibility trial of a lifestyle intervention (Motiv8) + TAU compared with TAU (+ Motiv8 waitlist) for adults on secure mental health units will be conducted. Thirty-two people (4 cohorts) will be recruited from secure services in Greater Manchester Mental Health NHS Foundation Trust. Participants will be randomly allocated to Motiv8 or TAU + Motiv8 waitlist. All participants will receive Motiv8 during the trial. Assessor-blinded physical/mental health and lifestyle assessments will be conducted at baseline, 10 weeks (post-intervention/waitlist), and after 12 weeks (post-waitlist intervention/follow-up). Motiv8 is a multidisciplinary intervention including exercise sessions, cooking/nutrition classes, physical health education, psychology sessions, sleep hygiene, peer support and medication review by pharmacy. A nested qualitative study will be conducted with a subsample of participants (*n* = 10) to explore their experiences of taking part. The analysis will focus on feasibility outcomes and tabulated success indicators of the study (e.g. Recruitment rates, retention rates, follow-up retention and response rates, attendance at sessions, the experience of involvement in the trial and delivery of the intervention, assessment of safety, development of a manualised intervention). Thematic analysis will be conducted through qualitative interviews. The analysis will aim to inform the development of a definitive trial.

**Ethics and dissemination:**

The trial has been granted ethical approval from the NHS Health Research Authority and adopted onto the UK Clinical Research Network Portfolio. Findings will be disseminated via peer-reviewed publications, professional and public networks, conferences and clinical services.

**Trial registration:**

ISRCTN13539285.

## Strengths and limitations of this study

### Strengths

- *Relevance of intervention.* There is a need to explore the feasibility and implementation of weight management interventions for people in secure forensic services, as research in this setting is scarce compared with the rest of the literature.
- *Multi-disciplinary intervention.* Motiv8 is a multi-disciplinary intervention which includes a multi-pronged approach to improving physical health (including nutrition, psychological guidance, exercise groups, occupational therapy input, pharmacy review and nursing input).
- *Peer support and user informed.* This is the first physical health intervention to be conducted in secure services which has been designed by service users, staff and academics and where service users play an active role in the delivery and facilitation of the intervention.
- *Data collection methods.* The study uses both quantitative and qualitative methods to collect data from a variety of sources including clinical notes, service user assessments/interviews, and staff consultations.

### Limitations

- *Small sample.* Due to resource and time constraints, the sample size for this pilot trial is small and consists of four cohorts of eight people (*n* = 32).
- *Mechanism of action.* It is unclear at this stage what the possible mechanisms of action may be for Motiv8 due to the multi-disciplinary approach.
- *Follow-up period.* Due to resource and time constraints, participants will only be followed up for 3 months after the end of the intervention, although proof of concept data will be collected at 6 and 9 months for the first two cohorts.
- *Lack of cultural diversity.* Only participants who have at least a basic understanding of English can take part as it will hinder their participation in the sessions and would not permit reliable estimates of the feasibility of outcome measures.
- *Waitlist control.* All participants will receive Motiv8 during the study which limits the scope of the follow-up assessments.

## Introduction

### Rationale

People with serious mental illness (SMI) have poor physical health [1]. They are more likely to develop cardiovascular disease and obesity, receive substandard physical health care, and live an unhealthy lifestyle, resulting in a 25-year reduction in life expectancy [1–3]. This issue has been labelled a ‘national scandal’, leading to increased calls to action for improving outcomes for people with SMI, such as the International Lancet Commission for physical health care in mental health services [4], and Public Health England (PHE) guidance to reduce health inequalities [5–8].

Individuals in secure mental health services are at even higher risk. Secure mental health services (or forensic inpatient units) treat and support people with SMI who may pose an imminent risk to themselves and others [9]. Secure services have a dual purpose; to treat mental illness and address offending behaviour and therefore, receive a quarter of the total mental health funding budget [9, 10]. Approximately 6000 people reside in secure services in the UK [5], and inpatient admissions usually exceed 5 years, with 20% of people staying for longer than 15 years [5, 11–13].

Rates of obesity in secure services have been shown to reach up to 70% of inpatient admissions [14], and correlations have been found between length of stay and weight gain [15]. Conditions such as cardiovascular disease and type 2 diabetes are more common in secure units than in generic inpatient units [14, 16–18]. People are more likely to live an inactive lifestyle, have high levels of adverse health behaviours, receive polypharmacy and high doses of antipsychotic medication [7, 19, 20]. Physical health is also affected by the ‘obesogenic’ nature of the inpatient environment, resulting in fewer opportunities to be active due to high levels of containment and restrictions on movement, reduced access to green space and community spaces, and increased access to unhealthy foods [21].

Updated World Health Organisation (WHO) guidelines recommend that increasing physical activity, reducing sedentary behaviour, and improving lifestyle have a beneficial impact on cardiometabolic health for people with SMI [22]. Despite a strong evidence-base showing physical health interventions improve mental and physical health across a range of mental health conditions [23–27], there have been relatively few well-conducted physical health studies in this setting. A previous National Health Service (NHS) commissioned review identified only one weight management randomised controlled trial (RCT), along with several small-scale uncontrolled programmes [18]. Previous approaches have included the use of digital technology for increasing activity levels (e.g. Wii fit, [28]), and nurse-led lifestyle interventions [29]. These initial studies have demonstrated some benefits to overall physical and mental health. However, existing interventions often fail to include control groups or comparators, standardised outcome measures, long-term follow-ups or use a multidisciplinary approach. Furthermore, they often have little input from service users throughout their development, consequently underrepresenting the ‘patient voice’. This is important as co-design is likely to increase the sustainability of the intervention and improve engagement by valuing patient experience throughout development [17, 30].

We aim to address this evidence gap by conducting a randomised trial of a weight management intervention (Motiv8). Motiv8 was co-developed, co-produced and is co-facilitated with service users. Four cohorts have taken place in an internal pilot where the programme was delivered as part of clinical care to people who wanted to take part. It was then reviewed and service users provided feedback on what they enjoyed and how they would improve it which has allowed us to develop and refine the intervention. Initial pilot data coming from consultations with 32 participants (unpublished) suggests a reduction in weight, change in waist circumference and improved cardiovascular fitness following the programme. Participants also reported increased energy, better sleep and improved sense of mental health and wellbeing. Work conducted to date has been an open trial, and therefore, no conclusions can be made as to the scientific feasibility of the programme under randomised conditions.

#### Aims and objectives

The primary aim is to conduct a randomised waitlist-controlled feasibility trial of a lifestyle intervention (Motiv8) + TAU for adults in secure mental health units, to investigate the acceptability, feasibility and potential effectiveness of this intervention to supplement standard secure care (see Table 1).

**Table 1:** Aims, objectives and outcomes

Research question/aim(s)	Objectives	Outcomes
Primary	To assess the acceptability and feasibility of the research trial, associated processes including the intervention, and assessments	Measured by: - Recruitment rates - Follow-up retention and questionnaire/outcome response rates - Attendance at sessions - Experience of involvement in the trial - Assessment of safety (SAEs) - Development of a manualised intervention
Secondary	Physical Health: Body composition, blood pressure, cardiovascular fitness, health status Mental Health: Wellbeing, depression, anxiety, negative symptoms Behavioural: Physical activity, occupational functioning, diet, sleep	Physical health measures: - BMI - BP - Hip/waist/chest/neck circumference - Fitness test Mental health measures: - WEMWBS - HADS - SNS Behavioural measures: - SIMPAQ - MOHOST - 24-h diet recall - PROMIS SD Short-Form - PROMIS SRI Short Form Measures to support economic evaluation: - EQ-5D-5L - ReQoL - Engagement in care - LUNSERS - Ward activity
Tertiary	Clarify training needs for delivering Motiv8 via a MDT care team, prior to the commencement of a definitive trial	- Qualitative interview - Adherence checklists - Feedback forms and interviews with facilitators - M-BacK Assessment and ESSEN-CES with clinical staff

*BMI* body max index, *BP* blood pressure, *ESSEN-CES* ESSEN Climate Evaluation Schema, *HADS* Hospital Anxiety and Depression Scale, *LUNSERS* Liverpool University Neuroleptic Side Effect Rating Scale, *M-Back* Metabolic-Barriers, Attitudes, Confidence and Knowledge Questionnaire, *MDT* multi-disciplinary team, *MOHOST* Model Of Human Occupation Screening Tool, *PROMIS SD* Patient Reported Outcome Measurement Information Centre Sleep Disturbance, *PROMIS SRI* Patient Reported Outcome Measurement Information Centre Sleep-Related Impairment, *ReQoL* Recovering Quality of Life, *SAEs* Serious Adverse Event, *SIMPAQ* Simple Physical Activity Questionnaire, *SNS* Self-evaluation of Negative Symptoms, *WEMWBS* Warwick Edinburgh Mental Wellbeing Scale

## Methods and analysis

### Trial design and flow chart

**Fig. 1:**
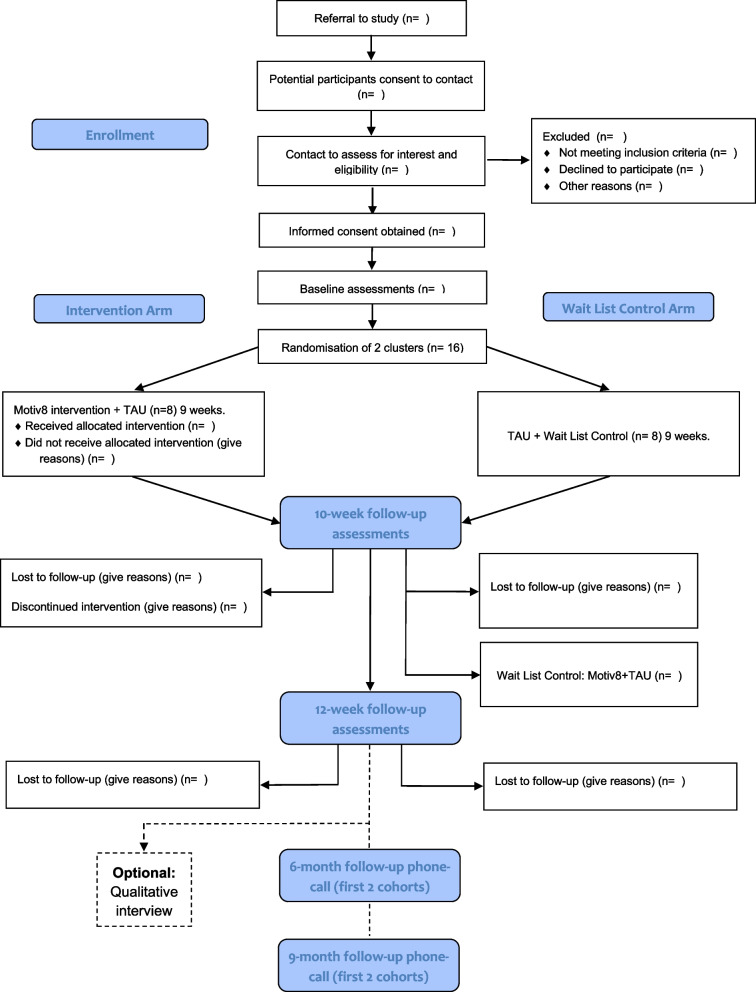
Flow chart

All procedures and conduct of this trial will be conducted in line with the CONSORT Extension to Randomised Controlled Trials [31]. The design is a prospective, single-blind, cluster-randomised controlled feasibility trial with two conditions; weight management intervention (Motiv8) plus treatment as usual (TAU), versus TAU waitlist control (with Motiv8 delivered after TAU). The study will take place in adult secure, forensic NHS mental health services. TAU will be measured throughout, and no treatment will be withheld from participants. Assessments will be completed at baseline (pre-intervention), 10 weeks (the week after participants finished Motiv8 or TAU), and again 12 weeks after the end of the intervention period (12-week follow-up). A nested qualitative study will explore the subjective experiences of taking part in Motiv8 and the acceptability of the intervention. See Fig. 1 for a summary of the trial design. The trial is prospectively registered on the ISRCTN registry: ISRCTN13539285 (ISRCTN-ISRCTN13539285: Motiv8: A weight management intervention for adults in secure mental health inpatient services). An independent Trial Steering Committee (TSC) and Experts by Experience Group have been established to provide ongoing guidance and oversight of the study.

### Participants

Participants will be current service users of low or medium-secure, adult forensic inpatient services in Greater Manchester Mental Health NHS Foundation Trust (GMMH NHS FT). The service provides individualised care and treatment for people with severe and enduring mental health disorders. We will aim to recruit a total of 32 participants forming four cohorts of Motiv8. Sample size is based on pragmatic limitations associated with the need to keep groups small due to the complex needs of service users, and the time constraints of funding. Following on from successful pilot work, clinical teams will be approached to identify eligible individuals to refer to the research team. The study will be advertised widely across the trust to service users and staff (such as through internal bulletins, social media, and recruitment materials). Clinicians will discuss the study with their service users and provide consent to contact. Researchers will provide potential participants with enough information to permit them to provide informed consent before taking part.

The inclusion and exclusion criteria are as follows:

#### Inclusion

- Adult inpatient (at least 18 years old) at mental health medium or low secure unit at GMMH NHS FT.
- Mental health diagnosis requiring treatment from secure services.
- Capacity to provide informed consent.

#### Exclusion

- Inability to provide informed consent in line with ethical requirements.
- Previous Motiv8 participant from the pilot work.
- Insufficient command of English/communication difficulties preventing engagement in written informed consent, the validity of research assessments or understanding of the programme.

Wards must have 8 people identified (maximum amount for each Motiv8 group) before randomisation. Each cohort will aim to contain people from the same ward to avoid conflict between patients and avoid contamination of the control groups; this decision is based on previous work in secure units, restrictions on movement and the internal pilot [12, 15, 17]. Individuals from the pilot phase and PPI consultations claimed being from the same unit is beneficial as it reduces anxiety being with people they know and avoids conflict between wards. Ongoing restrictions due to the COVID-19 pandemic also prevent mixing across different wards. In the instance that fewer than 8 people from the same ward are interested, or someone moves ward during the intervention period two wards may be combined to make up the cohort using a contingency plan developed within the study team. In line with principles of informed consent all participants will be made aware of their right to withdraw from the study at any point should they change their mind.

### Randomisation and blinding

Individuals will be cluster randomised by cohort, using minimisation to ensure balanced distribution. Following written consent, cohorts will be randomised using a free web-based system (www.sealedenvelope.com). Allocation will be communicated to the CI, study management team and facilitators but not research assistants, statistician or health economist. Participants will be informed of their randomisation status by letter or via clinicians, communicated via the administrator.

Blinding of allocation will be maintained for research assistants until all outcome measures for all subjects have been collected. Blindness will be maintained using a range of measures (e.g. separate offices for facilitators and researchers, protocols for answering phones, secretarial support). Unblinding will be communicated to the CI, and if possible, future assessments will be conducted by a blinded assessor. This may not always be possible due to the trial being cluster randomised, and therefore unblinding will occur on a cohort basis. Maintaining rater blindness to treatment allocation is crucial, and the TSC will be consulted in the instance that any unblinding occurs and implement corrective action if necessary.

### Assessments

**Fig. 2:**
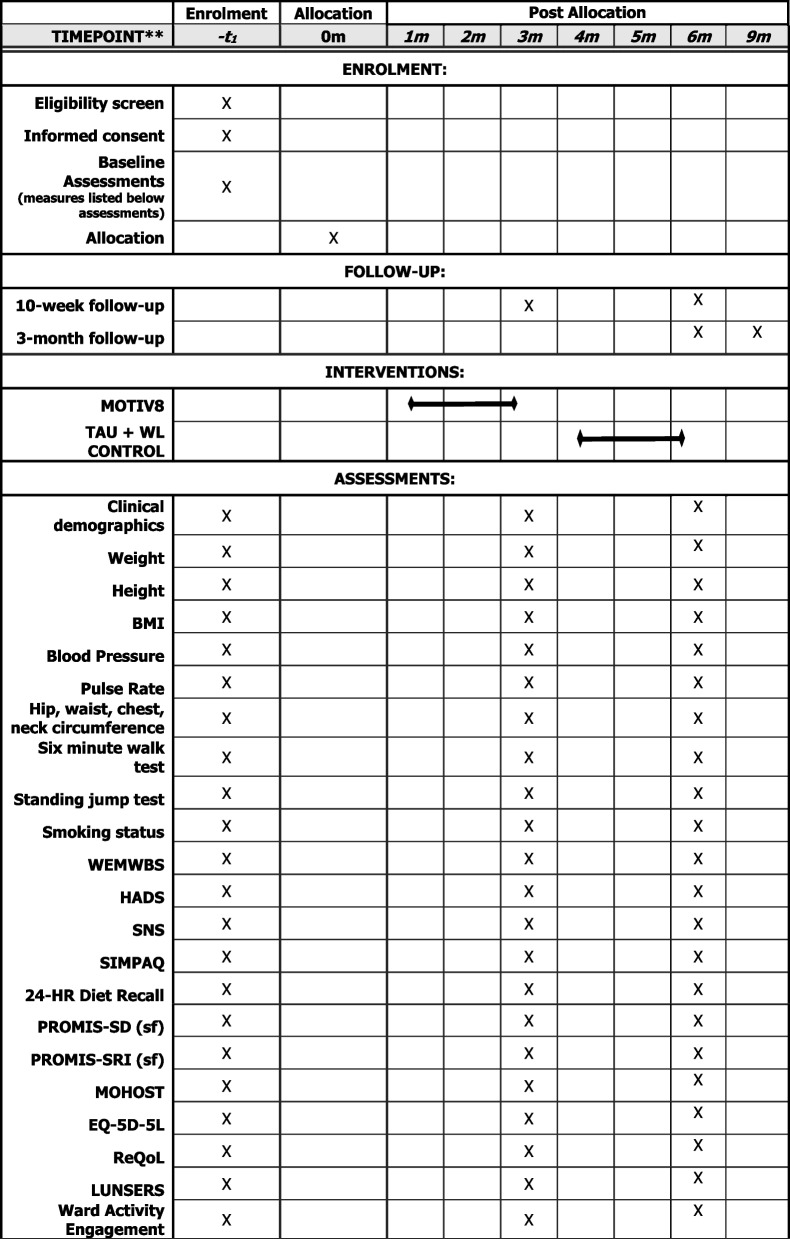
SPIRIT Diagram

Assessments will be conducted at three timepoints; baseline (pre-intervention/waitlist), week after intervention/waitlist (10 weeks) and follow-up (12 weeks after the end of the intervention). Basic sociodemographic and clinical information will be collected at baseline (e.g. age, gender, ethnicity, diagnoses, physical health conditions, duration of illness, length of admission). Clinically relevant information will also be collected at all three timepoints (e.g. medication, service use, treatment plan), (See Fig. 2).

The following measures will be collected:

- Physical health assessments
  BMI (Height/Weight), Blood Pressure, Pulse Rate, Hip/Waist/Chest/Neck Circumference, Cardiorespiratory fitness (using a vo2 sub max proxy test 6-min walk and standing jump test).
- Mental health assessments
  Warwick Edinburgh Mental Wellbeing Scale (WEMWBS; [32]), Depression and Anxiety (Hospital Anxiety and Depression Scale; [33]), Negative Symptoms (SNS; [34]).
- Behavioural assessments
  Physical Activity (SIMPAQ; [35]), Model of Human Occupation Screening Tool for nutrition sessions (MOHOST; [36]), 24-h diet recall [37], Sleep (PROMIS SD Short Form 8-item; PROMIS SRI Short form 4-item; [38–40].
- Measures to support future economic evaluation
  Health status (EQ-5D-5L; [41]), Quality of Life (ReQoL; [42]), the Liverpool University Neuroleptic Side Effect Rating Scale (LUNSERS; [43]), Engagement with ward activities and care.

Assessments are a mixture of self-report and researcher administered and may be collected over several sessions, with an aim of completing all within a week prior to or following the intervention. A nested qualitative study will be conducted with a subsample of participants (*n* = 10) after their final assessment to explore their experiences of taking part. Interview schedules will be developed with service user and carer input. To establish proof of concept of a longer follow-up period for a definitive study the first two cohorts will aim to be contacted after 6 and 9 months following the end of the intervention. Participants will be asked if it was a definitive trial whether they would be willing to complete assessments again.

### The Motiv8 intervention

Motiv8 is a 9-week, intensive programme co-developed with service users to improve the cardiovascular health of patients in secure inpatient units. The intervention aims to increase activity levels, improve diet, and use psychological guidance and techniques to maintain good physical health using goal-based techniques. Motiv8 is a multidisciplinary intervention encompassing several components to support physical health including exercise sessions, cooking/nutrition classes, physical health education, psychology sessions, sleep hygiene support, and a medication review (see Table 2 for an example schedule). Motiv8 will be facilitated and delivered by occupational therapists, dietitians, psychologists, pharmacists, physicians, exercise/sport professionals, nurses, and support workers. Trained service users assist with programme delivery to provide peer support and promote participant morale. An intervention booklet is provided consisting of resources, activities and prompts for goal setting/review of progress. A particular emphasis is placed on achievements and community, and participants will attend an awards ceremony upon completion.

**Table 2:** Example of Motiv8 intervention schedule

	**Exercise**	**Diet**	**Psychology group**	**Other**
Week 1	2 × sessions			Introduction to Motiv8
Week 2	3 × sessions	1 × session		
Week 3	3 × sessions	1 × session	1 × session	Physical health education session
Week 4	3 × sessions	1 × session		Sleep session
Week 5	3 × sessions	1 × session	1 × session	
Week 6	3 × sessions	1 × session		Pharmacy review
Week 7	3 × sessions	1 × session	1 × session	
Week 8	3 × sessions	1 × session		
Week 9	3 × sessions	1 × session		Award ceremony

The content of the sessions and all materials are agreed upon by the MDT team and research team. Exercise sessions are supervised and guided physical activity sessions lasting up to 1 h which include a mixture of cardio and strength-based exercises completed in onsite gym facilities and outdoor areas which gradually increase in difficulty. The cooking and diet sessions lasting 90 min are guided by group preferences and dietary requirements (e.g. halal, vegetarian). Recipes are selected by the group who are guided through the cooking process, having discussions about portion sizes and healthy food swaps. The group then engaged in a shared dining experience and sampled the food they cooked. Physical health education/sleep sessions are 1 h and interactive with a focus on providing education on looking after physical health, side effects of medication and ensuring positive sleep habits. Psychology sessions are interactive group sessions that are based on psychological theories of motivation and behaviour change, including problem-solving and goal setting.

Findings from successful pilot work across five cohorts indicated that Motiv8 may be feasible and beneficial to service users on secure units (unpublished). The purpose of this trial is to gather more information regarding the feasibility and potential efficacy of Motiv8 as a weight management intervention.

### Waitlist control group

Participants randomised to the waitlist control arm will receive their usual treatment and assessments at the same time points as those receiving Motiv8. Following the 10-week follow-up participants will receive Motiv8. Typical treatments for this group include pharmacological treatment, psychological therapy (group/individual), and various occupational therapy activities. They will be able to access gym facilities and be encouraged to eat well in line with usual care, and clinicians will be informed not to withhold any treatments whilst they await Motiv8. Any treatments received during the study will be recorded. After completing pre-/post-assessments individuals will receive Motiv8. This is an enhancement to routine care.

### Staff evaluation

Staff across the secure services will be asked to complete questionnaires to assess their attitudes and beliefs about physical health, and the ward atmosphere (ESSEN-Climate Evaluation Schema (ESSEN-CES; [44]), Metabolic-Barriers, Attitudes, Confidence and Knowledge Questionnaire (M-Back Questionnaire; [45]). Staff on non-participating wards will also be encouraged to complete questionnaires to allow comparison of the overall ward environment when not taking part in a physical health trial. Clinical staff will be encouraged to provide feedback in a specially designed questionnaire, or interviews after Motiv8 has taken place.

### Fidelity and facilitator evaluation

Fidelity assessments will be undertaken to see whether the intervention can be delivered according to protocol and inform a definitive trial. Adherence checklists specific to each component will be completed at the end of each session by facilitators. Assessment of facilitator performance will be conducted throughout the trial, during regular monitoring and supervision. Feedback will be collected from facilitators at the end of the trial and feedback strategy sessions will be held with facilitators and the research team. Intervention fidelity assessments, feedback and outcome measures will be used to inform a definitive trial.

### Analysis

A detailed Statistical Analysis Plan will be drafted by the study statistician prior to data analysis and a Health Economics Analysis Plan will be drafted by the study health economist. Quantitative analysis will be conducted according to intention-to-treat and reported according to CONSORT guidelines for cluster randomised pilot and feasibility studies [46], including the numbers of prospective participants who were approached, deemed eligible and consented. The number of participants who received their intended treatment (including which elements of it) will be reported.

There are no formal stopping rules for this study as the primary aim is to assess feasibility. The primary focus will be on tabulated and graphical summaries of key indicators of success of the study, e.g. recruitment, engagement, retention and satisfaction with the Motiv8 intervention (participant and facilitator). Where applicable these will be reported with a 95% confidence interval. All adverse events will be reported. We will summarise the baseline demographic and clinical characteristics of each cohort by trial arm. The completion rate of assessments will be reported, as well as descriptive characteristics such as mean (SD), median (IQR) or number (percentage).

To determine the potential utility of the Motiv8 intervention, an appropriate regression model will be fit to the data, using our intended primary outcome (weight), with a trial arm as a covariate controlling for gender and ward type. *P*-values will not be reported as this study is not designed to test effectiveness—instead, 70, 80 and 90% confidence intervals for the difference in weight between Motiv8 and TAU. Alternative proposed primary outcomes such as cardiovascular fitness, physical activity and well-being will be explored.

As Motiv8 is delivered in cohorts, a degree of intra-cohort correlation will exist in the outcomes. A sample size calculation for a definitive trial will require an estimate of the intra-cohort correlation. The correlation in this study will be investigated, but the number of cohorts is likely to be too small to obtain an accurate estimate. We will use descriptive statistics to inform the design of the economic components of the definitive trial, based on completion rate and summaries of relevant data (ReQOL, EQ-5D, service use and engagement, treatments).

Qualitative interviews will be audio-recorded and transcribed verbatim. Thematic analysis will be conducted according to the five-phase procedure described by Braun and Clarke [47]: familiarisation; initial code generation; searching and identifying themes; reviewing themes; and defining and naming themes.

### Patient and public involvement

Extensive PPI work underpins this study. Motiv8 has been co-developed and co-produced with service users and staff, from conception and ongoing iterative updates have been used to incorporate feedback from previous cohorts. Multiple discussion groups and consultations have been held with service users in secure services to refine the protocol and inform the design pre- and post-funding allocation. All research materials, such as information sheets, branding materials and recruitment leaflets have been co-produced with service user input. The core research team consists of investigators with lived experience and parent/carer representatives. PPI representatives will ensure the research is appropriate and sensitive to the needs of service users. People with lived experience will assist with the delivery of the intervention and provide peer support. Following the award of the grant, an independent expert by-experience group has been set up consisting of people who have previously used secure services. This group meets bimonthly and will continue to advise on all aspects of study progress (e.g. recruitment, analysis, and dissemination).

### Progression criteria

A red/amber/green criterion for progression to a full trial will be used, with a stop/refine/go approach. This will likely be recruitment > 80% (green), 60–79% (amber), < 60% (red) of planned target; adherence > 70% (green), 50–69% (amber), < 50% (red) attendance at planned sessions; retention within the study > 75% (green), 60–74% (amber), < 60% (red) completion of proposed primary outcome for the definitive trial (weight). Retention to the proposed primary outcome measure for a definitive trial (change in weight) will be monitored.

## Ethics and dissemination

### Ethics and dissemination

The trial has received Health Research Authority (HRA) approval (IRAS 299909) from the London–Bromley Research Ethics Committee (25th October 2021, 21/LO/0658). Local capacity and capability to deliver the research will be provided by the research department at the sponsoring organisation, Greater Manchester Mental Health NHS FT. Dissemination will occur with researchers, staff, service users and PPI representatives. Outputs and results of the trial will be published in open-access, peer-reviewed international journals where possible. Authorship will follow International Committee of Medical Journal Editors guidance (ICMJE | Recommendations | Defining the Role of Authors and Contributors). To increase reach, results will also be disseminated with the help of PPI input to non-academic audiences via media posts, blogs, newsletters and written summaries created with PPI groups. Summaries and updates will be presented to healthcare providers, managers and commissioners and shared via healthcare professional networks to ensure maximum reach. Results will be disseminated via local and international conferences and shared across social networks.

### Adverse events

Serious Adverse Events (SAEs) are untoward medical occurrences that result in death, are life-threatening, require inpatient hospitalisation or prolongation of existing hospitalisation or result in persistent or significant disability. For this study, other ‘important medical events’ are considered as adverse events such as serious violent incidents, or increased security status. SAEs are expected throughout the study period, such as incidents of self-harm, or violence and aggression. Exercise sessions form part of the intervention and therefore, adverse reactions such as muscle soreness and injury are more likely during the study period. Strategies to mitigate these risks have been incorporated into the study protocol (e.g. supervised sessions, graded increase in exercise). A trial standard operational procedure has been created to ensure all SAEs which are reported to the research team are assessed and categorised in line with HRA requirements. The research team, TSC, and experts by experience group will be made aware of any SAEs and will determine whether they are related to participation in the trial. Immediate safety concerns will be addressed as soon as possible with strategies put in place to prevent further risk to participants. Clinical notes will be reviewed at the end of the study period and any SAEs will be recorded if deemed relevant to participation. Adverse events will be reported in the final write-up of the trial where there is no risk of a confidentiality breach.

### Trial status

The trial begun recruitment in December 2021. Recruitment will occur in two stages (to allow recruitment of the four clusters on a ward-by-ward basis) and will be complete by approximately July 2022 for the main cohort. The staff sub-study begun recruitment in December 2021. The study has been granted an extension to September 2023. Final outcome data will be collected by August 2023 and analysis completed by September 2023. A trial paper with outcomes is expected to be submitted for publication by January 2024.

## Data Availability

Not applicable. A full dataset is not yet available for this work and data sharing is not yet available.
